# Hainan four‐eyed turtles actively select suitable stones to masquerade according to their own morphology

**DOI:** 10.1002/ece3.11693

**Published:** 2024-07-01

**Authors:** Hongmin Yu, Xinyi Deng, Fanrong Xiao, Haitao Shi

**Affiliations:** ^1^ Ministry of Education Key Laboratory for Ecology of Tropical Islands, Key Laboratory of Tropical Animal and Plant Ecology of Hainan Province, College of Life Sciences Hainan Normal University Haikou China; ^2^ Haikou No.1 Middle School Haikou China

**Keywords:** body shape, body size, camouflage, coloration, microhabitat selection

## Abstract

Masquerade is a form of camouflage in which animals use their body size, shape, and coloration to resemble inanimate objects in their environment to deceive predators. However, there is a lack of experimental evidence to show that animals actively choose objects that match these body parameters. To explore how the Hainan four‐eyed turtle, *Sacalia insulensis*, masquerades using suitable stones, we used indoor video surveillance technology to study the preferences of juvenile *S. insulensis* for stones of different sizes, shapes, and colors. The results indicated that under normal conditions, during the day, juvenile *S. insulensis* preferred larger oval or round stones, while at night, they preferred oval stones that were closer to their own size, with no significant preference for stone color during either time. When disturbed (by a researcher swinging their arm back and forth above the experimental setup every hour to mimic a predator), the turtles showed a preference for brown stones that were closer to their size and oval in shape. These findings suggest that juvenile *S. insulensis* prefer stones that resemble their carapace size and shape to masquerade when undisturbed, and that this preference is reinforced when they masquerade to reduce the risk of predation. The preference for stones that resemble their carapace color is significant only when there is a disturbance. To the best of our knowledge, this is the first study to provide evidence that vertebrates can selectively choose objects that resemble their own morphology for masquerading to reduce predation risk.

## Graphical abstract

This study aimed to explore the preferences of Hainan four‐eyed turtles, Sacalia insulensis, for suitable stones to enhance masquerade. Using indoor video surveillance technology, we found that under normal conditions, the turtles preferred larger oval or round brown stones. When disturbed, the turtles showed a preference for brown stones that were closer to their size and oval in shape.

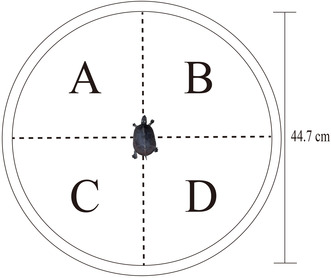

## 1 INTRODUCTION

Masquerade, as defined by Stevens and Merilaita (2009), is a novel camouflage strategy in which animals resemble inanimate objects found in their environment, such as stones, tree branches, and fallen leaves, allowing them to evade recognition by predators. Initially, this strategy was confused with mimicry (Wallace, 1889). However, masquerade is distinct from mimicry because it involves resembling nonliving objects or plants rather than other animals. For example, the larvae of the peppered moth caterpillar, *Selenia dentaria*, resemble tree branches in both appearance and coloration (Skelhorn, Rowland, & Ruxton, 2010), and cephalopods can change their body shape and patterns to resemble nearby objects (Panetta et al., 2017). Unlike animals using crypsis strategies that rely on the background matching of colors and patterns or disruptive coloration to avoid detection, masquerading animals must resemble both the coloration and morphology of inanimate objects, which, upon detection, lead predators to mistake them for inedible or uninteresting objects.

The predation risk for masquerading animals is contingent on their ability to be recognized by potential predators. The potential for recognition is influenced not only by the cognitive experience of predators but also by the inherent characteristics of masquerading animals, such as their body size, shape, and coloration (Mänd et al., 2007; Troscianko et al., 2013). Body size plays a significant role in the selection and effectiveness of masquerade strategies in animals. During the lifespan of lepidopteran larvae and invertebrates, they adopt masquerade strategies but only achieve protection when their size is similar to that of the selected object. Otherwise, they may resort to alternative defense strategies, such as aposematism (Futahashi & Fujiwara, 2008; Gaitonde et al., 2018; Postema, 2022; Valkonen et al., 2014; Yu et al., 2022).

Resembling the shape of the object is a fundamental requirement for the success of masquerade, as observed in stick insects masquerading as twigs and some caterpillars masquerading as bird droppings, which are the most common examples of masquerading organisms. When at rest, masquerading stick insects attach to tree trunks and maintain a certain angle to resemble branches (Dockery et al., 2009), whereas masquerading caterpillars contort their bodies to appear more like a collection of bird droppings when on leaves (Suzuki & Sakurai, 2015). This indicates that the body shape of masquerading animals not only has to roughly resemble that of the object but can also be altered through postural adjustments to allow it which resemble the object more closely.

The importance of body coloration resembling the coloration of an object is evident. In various Lepidopteran larvae that masquerade as twigs, both polymorphisms and polyphenisms are present. Their body coloration changes in response to different diets and the light conditions of their feeding environment (Greene, 1989; Skelhorn, Rowland, & Ruxton, 2010). For example, the body color of the American peppered moth caterpillar *Biston betularia cognataria* changes with the color of light in its feeding environment. On birch and willow trees, the caterpillars' coloration matches the twigs of these respective tree species (Noor et al., 2008). This demonstrates that masquerading animals alter their phenotype to enhance their masquerading effect, striving to closely resemble an object. However, such alterations incur high costs, and, compared to cryptic strategies, phenotypic traits exert stronger constraints on masquerading (Higginson et al., 2012). Therefore, masquerading animals are more likely to enhance their resemblance to an object by actively seeking suitable microhabitats that match their phenotypic characteristics (Skelhorn, Rowland, Speed, De Wert, et al., 2010; Skelhorn & Ruxton, 2013).

However, the proactive selection of microhabitats by animals has received little experimental research attention within the broader context of masquerade and has not been fully considered a key aspect of masquerade (Caro & Koneru, 2021; Stevens & Ruxton, 2019). In invertebrates, Skelhorn et al. (2010, 2013) demonstrated that body size influences microhabitats selection by animals. They found that different‐sized caterpillar larvae exhibit size preferences for branches, and when larvae are similar in length to branches, predators require more time to find them. Cooper Jr and Sherbrooke (2012) were the first to observe proactive microhabitat selection to enhance masquerading in vertebrates. They found that the round‐tailed horned lizard, *Phrynosoma modestum*, preferentially selected rocky habitats with rocks roughly the same size as the lizards, rather than bare sand habitats, and that masquerading efficiency was higher in habitats with rocks. Furthermore, Nafus et al. (2015) quantified rock size and found that juvenile desert tortoises, *Gopherus agassizii*, preferentially selected rocks in their habitat that were equal to or slightly larger than their body size. Thus, body size influences the preferences of masquerading animals for particular microhabitats. However, the impact of shape and color similarity on the selection of microhabitats by masquerading animals has yet to be experimentally validated in both invertebrates and vertebrates.

Behavioral experiments using captive‐bred animals may not fully reflect the behavioral patterns of wild animals in natural environments, especially considering that they have hardly experienced predation, which could lead to differences or changes in their antipredatory behavior compared to that of their wild counterparts (Martin, 2014). However, it should be noted that even prey without predation experience will show innate, instinctive responses to predator signals (Sündermann et al., 2008; Weiss et al., 2015). Currently, we still lack sufficient research records on the extent to which the masquerade behavior observed in laboratory environments reflects the habitat selection of animals in wild environments, especially for predator‐naive animals (Nafus et al., 2015).

The Hainan four‐eyed turtle, *Sacalia insulensis* (Testudines: Geoemydidae), recently revalidated from synonymy with *S. quadriocellata* and other *Sacalia* species, primarily inhabits mountain streams with dense boulders (Lin et al., 2018, 2022; Shi et al., 2008). In a stream in Luoma Village, Wanling Town, Qiongzhong County, Hainan Province, China, it was observed that the population density of *S. insulensis* inhabiting the midstream section of the river was significantly higher than that in the upstream and downstream sections. Based on shape description methods, the body shape of adult *S. insulensis* most closely resembled the morphology of cobblestones in the microhabitat substrate in the middle reaches of a river, leading to a lower rate of human detection than for *S. insulensis* individuals in other sections of the river (Xiao et al., 2021). This suggests that *S. insulensis* is an ideal experimental subject for studying the proactive selection of microhabitats by masquerading animals.

However, existing research has been limited to quantifying the shape similarity of adult turtles randomly placed in different sections of the stream and has not explored the process of their autonomous selection of different objects. Hypothesis testing regarding the species' preference for microhabitats has not been conducted, and further investigation is needed to establish the relationship between the proactive habitat selection behavior of masquerading animals and their body size, body shape, and coloration.

In this study, we focused on juvenile *S. insulensis*, which faces significant predation pressure, as the research subjects. We designed three experimental groups based on body size, shape, and coloration characteristics to observe their preferences for stones of different sizes, colors, and shapes under normal day–night conditions and human disturbance. *S. insulensis* exhibits distinct diurnal and nocturnal behavioral differences. Under laboratory conditions, captive‐bred *S. insulensis* rest predominantly during the day and become more active at night, primarily foraging (Liu et al., 2009). Investigating their stone preferences in these different behavioral states is crucial for understanding their masquerade protection mechanisms. We predicted that *S. insulensis* would preferentially select stones resembling their own characteristics as habitats and that their choice of stone features may be more consistent with their body size, coloration, and shape when affected by human disturbance, including normal night conditions. We aimed to verify the role of external animal characteristics in habitat selection and thus the understanding of turtle masquerading. We are also committed to further assessing the consistency between the anti‐predatory behavior of captive‐bred animals and microhabitat selection in the wild as well as to exploring its application value in conservation management.

## 2 MATERIALS AND METHODS

### 2.1 Experimental animals

The nine juvenile *S. insulensis* used in this study were bred at the College of Life Sciences, Hainan Normal University. They were laboratory‐bred descendants from individuals sourced at a stream in Luoma Village, Wanling Town, Qiongzhong County, Hainan Province, China. They had an average carapace length (CL) of 7.23 cm, average carapace width (CW) of 5.34 cm, average carapace height (CH) of 2.56 cm, and average carapace width‐to‐carapace length ratio of 0.74.

### 2.2 Experimental materials and experimental platform setup

**FIGURE 1:**
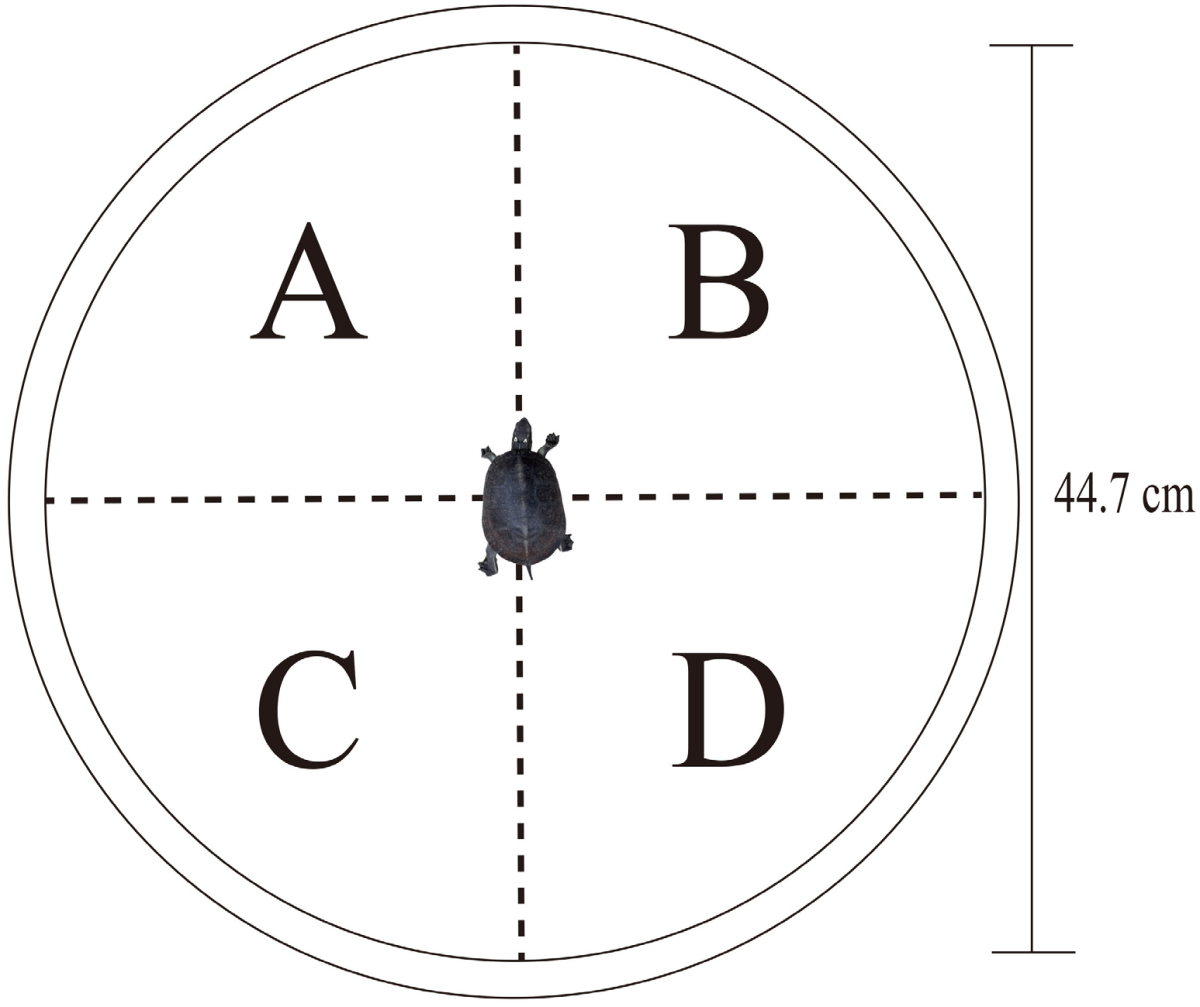
Schematic diagram of the experimental setup. In three parallel experiments, area A was filled with large stones, elliptical stones, and black stones; area B was filled with medium‐sized stones, circular stones, and brown stones; area C was filled with small stones, rectangular stones, and gray stones; area D was left blank for the stone size and shape selection experiment, while white stones were used for the stone color selection experiment.

The entire experiment was conducted under 24‐h video surveillance, and the whole process was recorded using cameras. The experimental setup was placed within a rectangular experimental pool, with dimensions of 1.45 m × 0.65 m and a depth of 0.55 m, in an indoor laboratory, with temperature maintained within a range of 19–22°C. To accommodate the full range of activities of *S. insulensis*, the size of the experimental setup was sufficient to allow them to move freely around any given stone, thereby accurately demonstrating their natural selection behavior. A circular plastic basin with a diameter of 44.7 cm was placed in the pool. Nylon ropes were used to cross‐fix the area above the plastic basin, dividing it into four equal‐sized areas for the placement of various stone types (Figure 1). The stones were crafted from polymer clay, which is odorless, environmentally friendly, and nontoxic, ensuring the safety of juvenile *S. insulensis* during the experimental process. Moreover, this material offers high extensibility, plasticity, and versatility in color, facilitating the creation of various types of stones as required for this experiment.

Prior to each experiment, the appropriately colored polymer clay was kneaded and molded into the desired stone shape for a single experimental session. The molded clay stones were then placed in an oven for low‐temperature baking and curing. The duration of baking varied from 25 to 40 min depending on stone size. Careful attention was paid to avoid overbaking, which could lead to fracture, or underbaking, which would result in insufficient hardness and subsequent deformation upon immersion in water. Once the baked polymer clay stones were completely dried and their forms were stabilized, the experiments commenced. Before each experimental trial, the stones were arranged to ensure equal spacing between them. Approximately 1.25 L of water was then poured into the apparatus, with the water level covering the stones and the entire *S. insulensis* carapace (approximately 2.5 cm deep). During each experiment, a single turtle was released into the indoor experimental setup. The turtles were fed turtle food (LIFELINE, Zhejiang Dongyang Baiying Pet Food Co., Ltd) and some green vegetables prior to the experiments, with no feeding during the experimental process to minimize potential interference from external factors.

### 2.3 Experimental design

Three sets of experiments were conducted with varying stone sizes, shapes, and colors as the independent variables. All three sets of experiments consisted of two treatments: normal day‐night conditions and disturbance conditions. The experiment was conducted from April 2018 to April 2019. The order of experiments for each turtle was randomized, and the disturbance experiment was repeated twice.

#### 2.3.1 Stone size selection experiment

Four equally sized areas were designated within the circular plastic basin experimental setup. Three types of stones of different lengths were arranged for turtle selection according to the size of juvenile *S. insulensis*. The large stones had a length of 10 cm, exceeding the average CL by approximately 1.42 times. The medium‐sized stones had a length of 7 cm, which closely approximates the average CL. The small stones had a length of 5 cm, which was smaller than the average CL by approximately 1.42 times. The height of each stone type was fixed at approximately 2.5 cm, which was approximately equal to the turtle's average CH. All stones were colored brown to resemble the color of turtle carapaces. An additional blank area was used as the control group.

#### 2.3.2 Stone shape selection experiment

In accordance with the shell shape of juvenile *S. insulensis*, three types of stones with distinct shapes were placed within the respective experimental setups. The first type resembled an ellipse with a short axis of 5 cm and a long axis of 7 cm, resulting in a short‐to‐long‐axis ratio of 0.71. The second was a circular stone with a diameter of 5 cm, which matched the average carapace width of the turtle. The third type consisted of rectangular stones with a width of 5 cm and a length slightly exceeding the average carapace length. All stones of each shape type were colored brown to closely resemble the coloration of the turtles' carapace. A blank area was designated as the control group.

#### 2.3.3 Stone color selection experiment

Four different colors of stones, black, brown, white, and gray, were distributed evenly in the four equally sized areas within the circular plastic basin for the turtles to choose from. Each color type used corresponds to a specific reference. Brown was selected to resemble the color of the turtle carapace and closely resembles the color of some stones in their preferred habitat. Black represents the natural large stones found in their habitat, gray represents the lighter stones, and white stones were used as the control group. The color of the four types of polymer clay was in their original, unaltered state as purchased, with no additional pigments. Upon visual inspection, its color appears similar to that of natural stones in the environment. Each type of colored stone had identical dimensions: a length of 7 cm, width of 5 cm, and height of 2.5 cm. All the stones were elliptical, resembling the approximate size of juvenile *S. insulensis*, ensuring consistency across experimental conditions.

#### 2.3.4 Normal day and night observation experiments

The normal condition experiment involved monitoring the camouflage behavior of turtles in the absence of predators. The experiment ran from 6:00 on the first day to 6:00 on the following day. The diurnal period was defined as from 8:00 to 20:00, and the nocturnal period was defined as from 20:00 to 8:00 the following day. The entire process was recorded using a video surveillance system to capture all movements of the turtle within the setup. Subsequent analysis of the experimental footage was performed utilizing instantaneous sampling, with observations made every 10 min to record the turtle's current position during the experiment, thereby assessing its preference for the available stones at each moment. Turtles used for the experiment were introduced to the setup 1 day in advance to allow acclimatization, followed by a 72‐h experimental period. During this acclimatization period, there were no stones or other objects in the tank.

#### 2.3.5 Human disturbance experiment

We simulated human predation activities to observe changes in the turtle's masquerading behavior. The disturbance experiment commenced at 8:00 and ended at 20:00. Throughout this period, the turtles were disturbed hourly. The specific method involved a researcher swinging the arm back and forth for approximately 20 s above the experimental setup every hour to simulate human predation activities. We conducted a total of 13 disturbances. Through video surveillance, observations of the turtles' reactions within the area after being disturbed were recorded, including their positions before the disturbance and when the disturbance ceased. At other times, the turtles were free to move, and their positions were not recorded. Each turtle underwent two repeated experiments to ensure the reliability of the results. Throughout the experiment, the masquerade behavior of *S. insulensis* was determined by their autonomous choices, without any subjective control over their direction by the observer. Prior to each experiment, the turtles were allowed to acclimatize to the experimental environment for 1 day, ensuring that they exhibited relatively authentic instinctive responses to sudden predator invasions.

### 2.4 Statistical analysis

Using the experimental monitoring footage, we recorded the number of times juvenile *S. insulensis* was found on stones of different colors, sizes, and shapes, or in blank areas. Specific situations encountered during the experiment were statistically analyzed according to the following principles: if a turtle was found between two types of stones, the stone with the larger proportion within the nylon rope boundary was considered its final choice. If a turtle was at the boundary position, its choice was determined according to the direction of its head. Considering the impact of day and night on turtle activity, the results of the observation experiment were recorded separately for day time and night time. All statistical procedures were conducted and generated in R 4.3.0 (R Core Team, 2023). Statistical analysis was performed utilizing a maximum likelihood *G*‐test and applying a Bonferroni‐type correction, alpha was set to 0.05 (0.0167 after Bonferroni correction). All graphics were created using the ggplot2 package (Wickham, 2016) available in R 4.3.0 (R Core Team, 2023).

### 2.5 Ethics statement

All experiments were performed in strict accordance with the guidelines of the Animal Research Ethics Committee of the Hainan Provincial Education Centre for Ecology and Environment, Hainan Normal University (HNECEE‐2011‐003), which conforms to the Law of the People's Republic of China.

## 3 RESULTS

### 3.1 Stone size selection

**FIGURE 2:**
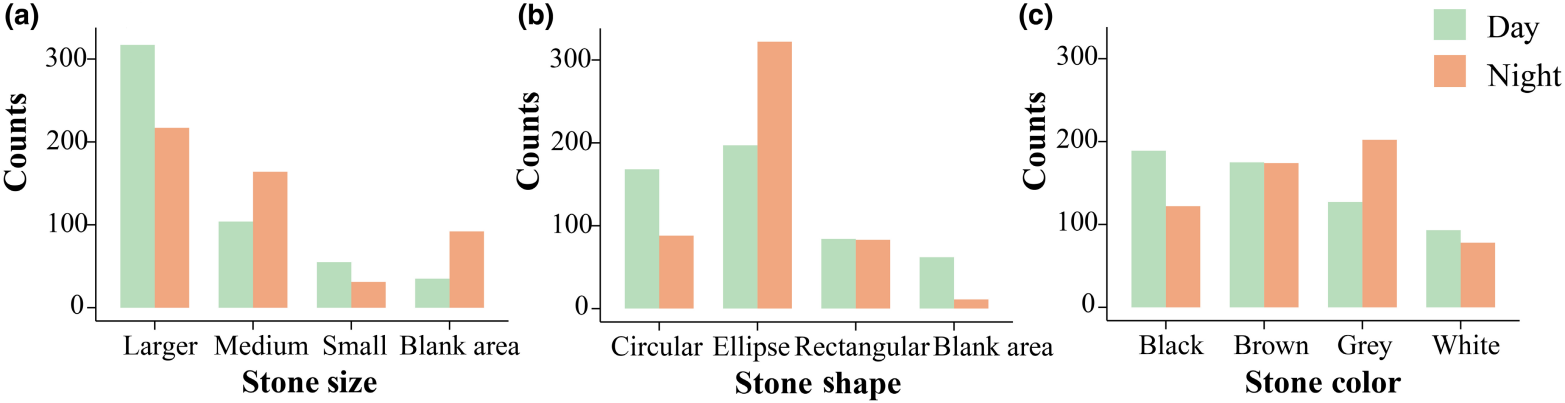
Preference of *Sacalia insulensis* turtles toward stones of different sizes (a), shapes (b), and colors (c) during the day and at night.

Our results showed significant differences in the frequencies of *S. insulensis* juveniles found on stones of different sizes between day and night (*G* test = 65.643, df = 3, *p <* .001) (Figure 2a). During the day, juveniles significantly preferred larger stones (62.04%, *G* test = 350.08, df = 3, *p <* .001), whereas at night, they preferred both large‐ (43.06%) and medium‐sized (32.54%) stones (*G* test = 177.58, df = 3, *p <* .001).

**FIGURE 3:**
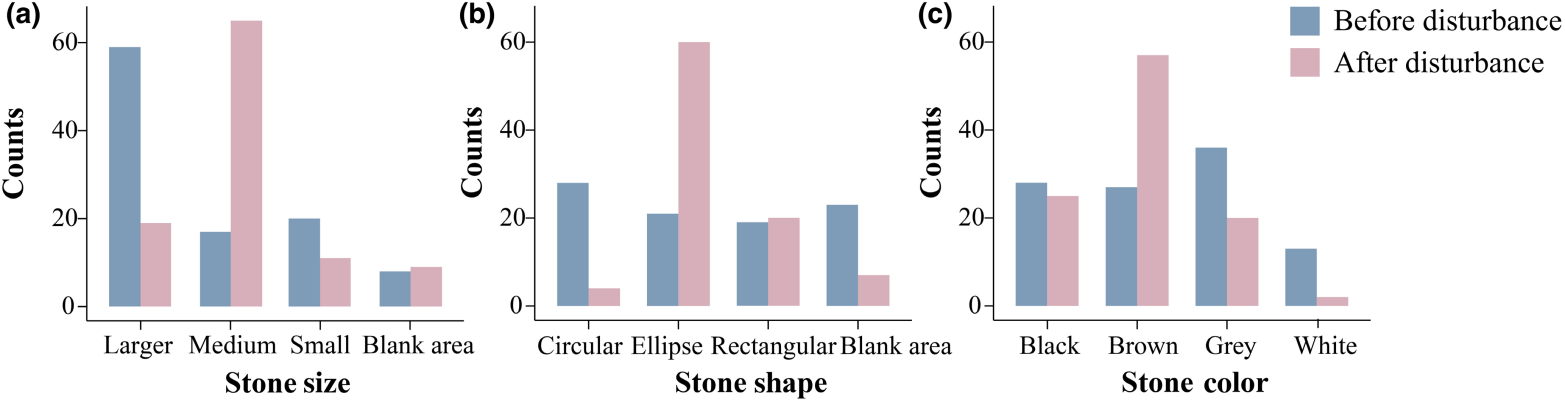
Preference of *Sacalia insulensis* turtles toward stones of different sizes (a), shapes (b), and colors (c) before and after disturbance conditions in daylight.

Significant differences were observed in the frequency of juvenile Hainan four‐eyed turtles found on stones of different sizes before and after predator disturbance (*G* test = 54.206, df = 3, *p <* .001) (Figure 3a). Before the disturbance, juveniles more frequently preferred large‐ (56.73%) and small‐sized (19.23%) stones (*G* test = 52.895, df = 3, *p <* .001). After the disturbance, their frequency of occurrence on medium‐sized stones (62.5%) increased while that large‐ (18.27%) and small‐sized (10.58%) stones decreased (*G* test = 69.179, df = 3, *p <* .001). This indicates that human disturbance significantly influenced stone size selection by *S. insulensis* juveniles.

### 3.2 Stone shape selection

There were significant differences in the frequency of *S. insulensis* juveniles found on stones of different shapes between day and night (*G* test = 95.096, df = 3, *p <* .001) (Figure 2b). During the day, the juveniles significantly preferred circular (32.88%) and elliptical (38.55%) stones (*G* test = 102.6, df = 3, *p <* .001), whereas at night, they preferred elliptical stones (63.89%), with a significant decrease in their occurrence on circular stones and open ground (*G* test = 418.13, df = 3, *p <* .001).

Significant differences were observed in the frequency of juvenile *S. insulensis* found on stones of different shapes before and after disturbance (*G* test = 48.847, df = 3, *p <* .001) (Figure 3b). Before the disturbance, the juveniles showed no significant differences in distribution on stones of various shapes (circular stones 30.77%, elliptical stones 23.08%, rectangle stones 20.88%, open ground 25.27%) (*G* test = 1.924, df = 3, *p* = .5883). After the disturbance, they showed a greater preference for elliptical stones (65.93%) that resembled their own body shape, with a significant decrease in their preference for circular stones (4.39%) and open ground (7.69%) (*G* test = 80.813, df = 3, *p <* .001). This indicates that human disturbance significantly influenced the selection of stone shapes by juvenile *S. insulensis*.

### 3.3 Stone color selection

The frequency of *S. insulensis* juveniles found on stones of different colors showed significant differences between day and night (*G* test = 33.062, df = 3, *p <* .001) (Figure 2c). White stones (15.92%) were the least frequented by *S. insulensis* juveniles during both the day and night. During the day, juveniles preferred black (32.36%) and brown (29.97%) stones (*G* test = 41.69, df = 3, *p <* .001), whereas at night, they preferred brown (30.21%) and gray (35.07%) stones (*G* test = 66.494, df = 3, *p <* .001). Despite the differences in preference between day and night, juveniles *S. insulensis* consistently showed a preference for brown stones.

Statistical analysis of disturbance experiment results revealed significant changes in the choice of stone color before and after the disturbance (*G* test = 24.774, df = 3, *p <* .001) (Figure 3c). Before the disturbance, fewer juveniles chose white stones (12.5%) and instead showed a preference for black (26.92%), brown (25.96%), and gray stones (34.62%) (*G* test = 11.597, df = 3, *p <* .001). However, after the disturbance, juvenile *S. insulensis* showed a greater tendency to choose brown stones (54.81%) (*G* test = 66.769, df = 3, *p <* .001).

## 4 DISCUSSION

Our results were largely consistent with our predictions, demonstrating that juvenile *S. insulensis* turtles exhibit distinct habitat selection behaviors. In the presence of human disturbances, turtles actively choose areas with stones resembling their own characteristics, namely, their body size, body shape, and coloration. We propose that this behavior is aimed at maintaining a better masquerade effect.

Under normal circumstances, juvenile *S. insulensis* exhibited a preference for stones that resemble their own size or larger, which is consistent with the findings of previous studies on peppered moth caterpillars, round‐tailed horned lizards, and desert tortoises (Cooper Jr & Sherbrooke, 2012; Nafus et al., 2015; Skelhorn & Ruxton, 2013). Following human disturbances, their preference for stones that are similar in size to themselves becomes particularly pronounced, indicating that body size is an important factor influencing active microhabitat selection by *S. insulensis* turtles. During normal day–night observation experiments, the preference of juvenile *S. insulensis* for large rocks significantly decreased at night, while their selection of medium‐sized rocks, comparable to their own size, notably increased. This behavior might be attributed to the circadian rhythms of captive‐bred *S. insulensis*, which spend most of the day resting and become more active in foraging at night (Liu et al., 2009). During the day, choosing larger rocks for habitation provides dual protection through avoidance and masquerade. At night, the dependence on masquerade for protection becomes more pronounced, and the low light conditions make it more difficult for predators to accurately detect and identify the turtles, enhancing their masquerade efficiency.

Previous studies have shown that the similarity in shape between adult *S. insulensis* and river stones is positively correlated with masquerading efficiency (Xiao et al., 2021). Our research further confirms this as, during nighttime feeding periods and in the presence of human disturbances (Liu et al., 2009), juveniles actively chose elliptical stones that resemble their own body shape for habitat activities. Additionally, although rectangular stones do not perfectly match their body shape, they may provide other resources such as shading or resting opportunities, which may explain why the frequency of turtles in areas with rectangular stones was lower but more consistent. Overall, the strong preference of *S. insulensis* for elliptical stones suggests that the similarity of body shape to that of the model object is a major factor in habitat selection decisions.

Juvenile *S. insulensis* exhibited a relatively stable preference for brown stones, which resemble their own carapace color, during both the day and night phases, and this preference was significantly enhanced under human disturbance. This indicates that, regardless of predation risk, *S. insulensis* actively seek habitats containing objects similar in color to their own bodies to better conceal and blend into a rocky environment, thereby gaining better masquerade protection. However, there is also a strong preference for black and gray rocks, suggesting that color may not be a significant constraint for the turtles' masquerade effectiveness. This could be due to the natural variety of rock colors in the natural environment, which is also perceived by predators. Predator cognition may play a more crucial role than sensory capabilities in driving the evolution of masquerade (Skelhorn, Rowland, Speed, & Ruxton, 2010). Even if the masquerade animal's color matches its model perfectly, predators with enhanced cognition and experience can better recognize masquerade animals (Skelhorn & Rowe, 2016; Stoddard, 2012). This may reduce the selection pressure on sensory‐driven color matching, allowing greater flexibility in microhabitat selection based on color. However, the extent to which body color similarity affects the turtles' masquerade efficacy requires further research. Integrating predator vision models, cognitive process models, and predation experiments will provide valuable insights into this matter.

In summary, under normal diurnal conditions, *S. insulensis* exhibits flexibility in its preferences for the size, shape, and color of stones during the day. However, at night, it shows a significant preference for stones that closely match its own characteristics. This preference becomes particularly pronounced when there is human disturbance. Therefore, our study found that body size, shape, and coloration are important factors influencing the active habitat selection behavior of *S. insulensis*, to enhance masquerade, with the purpose of enhancing masquerade. This indicates that captive‐bred *S. insulensis* possess certain perceptual and cognitive abilities, allowing them to make active masquerade decisions. We believe that wild *S. insulensis* exhibits similar, if not stronger, microhabitat selection abilities. These findings provide valuable insights for the conservation and management of wild *S. insulensis* habitats. The present study highlights the importance of considering the interactions between behavior, physiology, and morphology in exploring the evolution of masquerade strategies, especially in regions with strong predation pressures, such as the tropics, where the evolution of multifaceted anti‐predator defense mechanisms may be particularly important.

However, we did not evaluate the masquerade effectiveness of *S. insulensis* against their preferred stones using a human recognition experiment as predators, which will be further investigated in subsequent studies. In addition, future research could delve into other behavioral aspects of *S. insulensis*. For instance, when faced with human disturbance, they may opt for short bursts of swimming, abruptly halting when encountering an ideal substrate to enhance their masquerade. Alternatively, they prioritize evading predation, thereby gliding through the water for longer distances before coming to a stop. Additionally, when the turtles are positioned among stones that most resemble their bodies, they may adjust their body orientation, such as by aligning parallel to the long axis of elliptical stones, to enhance masquerade. Understanding this could provide further insights into their adaptive behaviors in response to their surroundings. Furthermore, we have limited knowledge of how *S. insulensis* perceive their own phenotypic characteristics and compare them with surrounding objects. Further research is needed to explore how behavioral traits co‐evolve with morphological and physiological characteristics to better understand the ecology and evolution of antipredator adaptations. This requires studies on the visual perception and cognitive abilities of animals (Skelhorn & Rowe, 2016; Skelhorn, Rowland, & Ruxton, 2010; Uy et al., 2017).

## AUTHOR CONTRIBUTIONS

**Hongmin Yu:** Conceptualization (equal); data curation (equal); formal analysis (lead); methodology (lead); writing – original draft (lead); writing – review and editing (lead). **Xinyi Deng:** Data curation (lead); methodology (lead); writing – original draft (equal). **Fanrong Xiao:** Conceptualization (lead); funding acquisition (lead); writing – review and editing (equal). **Haitao Shi:** Conceptualization (equal); writing – review and editing (equal).

## CONFLICT OF INTEREST STATEMENT

The authors declare no conflicts of interest.

### OPEN RESEARCH BADGES

This article has earned an Open Data badge for making publicly available the digitally‐shareable data necessary to reproduce the reported results. The data is available at https://doi.org/10.5061/dryad.h70rxwsw.

## Supporting information


Tables S1–S6.

## Data Availability

The data are available in the supplementary files.
