# Supplementary material for: Hainan four‐eyed turtles actively select suitable stones to masquerade according to their own morphology
Source: Ecol Evol. 2024 Jul 1;14(7):e11693. doi: 10.1002/ece3.11693 (PMC11216812; doi:10.1002/ece3.11693)
Supplement: Supplementary file 1 — Tables S1–S6. [file ECE3-14-e11693-s001.docx]

Table 1 Counts and G-test results of Hainan four-eyed turtles on stones of different sizes

|  | Larger | Medium | Small | Blank area | *G* | *P* |
| --- | --- | --- | --- | --- | --- | --- |
| Daytime | 317 | 104 | 55 | 35 | 350.08 | ＜0.001 |
| Nighttime | 217 | 164 | 31 | 92 | 177.58 | ＜0.001 |
| Diurnal variation |  |  |  |  | 65.643 | ＜0.001 |

Table 2 Counts and G-test results of Hainan four-eyed turtles on stones of different sizes before and after disturbance

|  | Larger | Medium | Small | Blank area | *G* | *P* |
| --- | --- | --- | --- | --- | --- | --- |
| Before disturbance | 59 | 17 | 20 | 8 | 52.895 | <0.001 |
| After disturbance | 19 | 65 | 11 | 9 | 69.179 | ＜0.001 |
| Difference before and after disturbance |  |  |  |  | 54.206 | ＜0.001 |

Table 3 Counts and G-test results of Hainan four-eyed turtles on stones of different shapes

|  | Circular | Ellipse | Rectangular | Blank area | *G* | *P* |
| --- | --- | --- | --- | --- | --- | --- |
| Daytime | 168 | 197 | 84 | 62 | 102.6 | <0.001 |
| Nighttime | 88 | 322 | 83 | 11 | 418.13 | ＜0.001 |
| Diurnal variation |  |  |  |  | 95.096 | ＜0.001 |

Table 4 Counts and G-test results of Hainan four-eyed turtles on stones of different shapes before and after disturbance

|  | Circular | Ellipse | Rectangular | Blank area | *G* | *P* |
| --- | --- | --- | --- | --- | --- | --- |
| Before disturbance | 28 | 21 | 19 | 23 | 1.924 | = 0.5883 |
| After disturbance | 4 | 60 | 20 | 7 | 80.813 | <0.001 |
| Difference before and after disturbance |  |  |  |  | 48.847 | <0.001 |

Table 5 Counts and G-test results of Hainan four-eyed turtle on stones of different colors

|  | Black | Brown | Grey | White | *G* | *P* |
| --- | --- | --- | --- | --- | --- | --- |
| Daytime | 189 | 175 | 127 | 93 | 41.69 | ＜0.001 |
| Nighttime | 122 | 174 | 202 | 78 | 66.494 | ＜0.001 |
| Diurnal variation |  |  |  |  | 33.062 | ＜0.001 |

Table 6 Counts and G-test results of Hainan four-eyed turtles on stones of different colors before and after disturbance

|  | Black | Brown | Grey | White | *G* | *P* |
| --- | --- | --- | --- | --- | --- | --- |
| Before disturbance | 28 | 27 | 36 | 13 | 11.597 | ＜0.001 |
| After disturbance | 25 | 57 | 20 | 2 | 66.769 | ＜0.001 |
| Difference before and after disturbance |  |  |  |  | 24.774 | ＜0.001 |

1. All p-values have been Bonferroni corrected, with the significance level adjusted to 0.0167.
